# The Encapsulation of Lycopene in Nanoliposomes Enhances Its Protective Potential in Methotrexate-Induced Kidney Injury Model

**DOI:** 10.1155/2018/2627917

**Published:** 2018-03-13

**Authors:** Nenad Stojiljkovic, Sonja Ilic, Vladimir Jakovljevic, Nikola Stojanovic, Slavica Stojnev, Hristina Kocic, Marko Stojanovic, Gordana Kocic

**Affiliations:** ^1^Department of Physiology, Faculty of Medicine, University of Niš, Bulevar Zorana Ðinđića 81, 18000 Niš, Serbia; ^2^Department of Physiology, Faculty of Medical Sciences, University of Kragujevac, Svetozara Markovica 69, 34000 Kragujevac, Serbia; ^3^Department of Human Pathology, I.M. Sechenov First Moscow State Medical University, Moscow, Russia; ^4^Faculty of Medicine, University of Niš, Bulevar Zorana Ðinđića 81, 18000 Niš, Serbia; ^5^Department of Pathology, Faculty of Medicine, University of Niš, Bulevar Zorana Ðinđića 81, 18000 Niš, Serbia; ^6^Faculty of Medicine, University of Maribor, Magdalenski trg 5, SI-2000 Maribor, Slovenia; ^7^Department of Biochemistry, Faculty of Medicine, University of Niš, Bulevar Zorana Ðinđića 81, 18000 Niš, Serbia

## Abstract

Methotrexate is an antimetabolic drug with a myriad of serious side effects including nephrotoxicity, which presumably occurs due to oxidative tissue damage. Here, we evaluated the potential protective effect of lycopene, a potent antioxidant carotenoid, given in two different pharmaceutical forms in methotrexate-induced kidney damage in rats. Serum biochemical (urea and creatinine) and tissue oxidative damage markers and histopathological kidney changes were evaluated after systemic administration of both lycopene dissolved in corn oil and lycopene encapsulated in nanoliposomes. Similar to previous studies, single dose of methotrexate induced severe functional and morphological alterations of kidneys with cell desquamation, tubular vacuolation, and focal necrosis, which were followed by serum urea and creatinine increase and disturbances of tissue antioxidant status. Application of both forms of lycopene concomitantly with methotrexate ameliorated changes in serum urea and creatinine and oxidative damage markers and markedly reversed structural changes of kidney tissue. Moreover, animals that received lycopene in nanoliposome-encapsulated form showed higher degree of recovery than those treated with free lycopene form. The findings of this study indicate that treatment with nanoliposome-encapsulated lycopene comparing to lycopene in standard vehicle has an advantage as it more efficiently reduces methotrexate-induced kidney dysfunction.

## 1. Introduction

Methotrexate (MTX) is antimetabolic drug which is often used for the treatment of several autoimmune disorders such as rheumatoid arthritis, psoriasis, and various malignant tumors including lymphoblastic leukemia, lymphoma, osteosarcoma, breast cancer, and head and neck cancer [1]. Clinical use of MTX is limited due to its side effects that include bone marrow suppression, hepatotoxicity, nephrotoxicity, pulmonary fibrosis, and gastrointestinal mucosal damage. Since MTX is primarily excreted by kidneys [2], severity of nephrotoxicity depends on both the dose and frequency of MTX administration [3]. Similar to other agents with nephrotoxic effect, MTX-induced renal impairment is clinically followed by hematuria and increased levels of serum creatinine and urea in humans. These effects can be faithfully reproduced in experimental animals after single dose of MTX [4, 5].

Lycopene, a red-colored carotenoid, found mainly in tomatoes but also in other fruits and vegetables (watermelons, *Momordica cochinchinensis*, Spreng fruit, papayas, etc.), possesses a strong antioxidant activity. It protects cells against damage caused by free radicals with its reactive oxygen species (ROS) scavenging properties [6, 7]. It is proven to be more potent than similar antioxidants, like *β*-carotene and vitamin E [6, 8], largely due to its several conjugated double bonds. Besides the numerous beneficial properties (anti-inflammatory, anticancer, etc.), investigations revealed that lycopene has protective effect in animal models of nephrotoxicity [8, 9].

Nanoliposomes are bilayer lipid vesicles which can be encapsulated with various bioactive agents including medicaments, pharmaceuticals, nutritional supplements, antioxidants, polynucleotides, and polypeptides [10]. Nanoliposomes have the potential to increase solubility and bioavailability, *in vitro* and *in vivo* stability, improve time-controlled drug releasing, minimize concentrations required for optimum therapeutic efficacy, enable cell-specific targeting, and decrease adverse effects of drugs on healthy cells and tissues [11, 12]. In the case of lycopene, formulation with nanoliposomes could prevent its rapid interaction with highly reactive compounds, like plasma proteins or metal ions, allowing it to reach the targeted damaged tissue. Moreover, using nanoliposomes as carriers to incorporate lycopene into membranes might be an initial step in cell prevention as it is proposed that damage of cell membrane by ROS causes the onset of number of pathological events leading to oxidative injury [13].

Since, to the best of our knowledge, no study exists which shows potential of using lycopene in this new pharmaceutical formulation to treat drug-induced tissue injury, we aimed at evaluating characteristics of the nanoliposome encapsulation and efficacy of this preparation in preventing/ameliorating methotrexate-induced kidney injury. To achieve this, we first measured in vitro properties of encapsulated lycopene and then compared its efficacy with free form lycopene through the evaluation of changes in serum and tissue oxidative stress markers and quantification of kidney tissue damages induced by MTX.

## 2. Materials and Methods

### 2.1. Drugs and Chemicals

Methotrexate was obtained from EBEWE Pharma (Ges.m.b.H.NFG.KG, Austria), while ketamine (Ketamidor 10%) was purchased from Richter Pharma (AG, Wels, Austria). Lycopene was purchased from Sigma-Aldrich (St. Louis, USA) and all other used chemicals were obtained from either Sigma-Aldrich (St. Louis, USA) or Carl Roth (Karlsruhe, Germany).

### 2.2. Experimental Protocol In Vitro

#### 2.2.1. Nanoliposomes Encapsulation with Lycopene

The 10% solution of phospholipid nanoparticles, in a form of nanospheres, purchased from Nattermann Phospholipids (Germany), was encapsulated by lycopene at the concentration of 4 mg/ml. Encapsulated nanoparticles were isolated by centrifugation at 6500*g* for 30 min at 4°C according to the method of Kocic et al. [13].

#### 2.2.2. Efficacy of Lycopene Encapsulation

The efficacy of encapsulation was determined based on a method previously described in great detail [14]. Briefly, after mixing the lycopene-loaded liposomes and petroleum ether (3 ml), the mixture was further vortexed for 3 min followed by a centrifugation (2000 rpm, 5 min). The upper layer was separated and its absorbance was measured at 470 nm (V-1800 Shimadzu spectrophotometer). The encapsulation efficacy (%) was calculated as (amount of incorporated lycopene)/(initial amount of added lycopene) × 100.

#### 2.2.3. Sustained Release of Encapsulated Lycopene

The mixture of lycopene nanoparticles with phosphate buffer saline was incubated at 37°C for 24 h from which the aliquots were taken in order to measure the release of lycopene from nanoliposomes. At the defined time points (0, 1, 2, 4, 8, 12, and 24 h), the aliquots were taken and diluted in petroleum ether in order to measure their absorbance at 470 nm and quantify the amount of free lycopene present. The content of lycopene was calculated using a standard curve.

#### 2.2.4. pH Dependent Stability of Encapsulated Lycopene

The stability of free and encapsulated lycopene in different pH buffers (6.5, 7.4, 8.0, and 9.0) was studied following previously described method [15]. The mixture containing either free or encapsulated lycopene and buffer solution with different pH values (1 : 10, *v*/*v*) was incubated at 37°C, from which the aliquots were taken after 0, 20, 40, 60, 120, and 180 minutes. The residual rate of lycopene present in the mixture at designated time point was determined spectrophotometrically at 470 nm using a standard curve for lycopene. Residual rate is the residual amount of lycopene expressed in percentages in relation to the initial amount of lycopene.

#### 2.2.5. Metal Ion Chelating Properties of Encapsulated Lycopene

The stability of formed lycopene nanoliposomes in the presence of different metal ions (K^+^, Ca^2+^, Mg^2+^, Al^3+^, or Cu^2+^) was evaluated according to the method described by Chen et al. [15]. The free/encapsulated lycopene was incubated with 1 mmol/l of metal solution for 2 h at 37°C. After the incubation period, the mixture was centrifuged in order to separate the formed lycopene-metal chelate. The obtained supernatant was mixed with petroleum ether, and the absorbance of the solution was measured at 470 nm in order to determine the residual rate of lycopene, where residual rate is the residual amount of lycopene expressed in percentages in relation to the initial amount of lycopene.

#### 2.2.6. Susceptibility of Encapsulated Lycopene to H_2_O_2_

After centrifugated nanoparticles were resuspended and both lycopene encapsulated and native nanoparticles as well as free lycopene were exposed to H_2_O_2_ for 30 min at 37°C [13]. The intensity of lipid peroxidation was determined using colorimetric method that involves thiobarbituric acid as a reagent [16].

### 2.3. Experimental Protocol In Vivo

#### 2.3.1. Animals and Housing

Forty-eight male Wistar rats (200–250 g) were divided into 8 groups of 6 animals and maintained under standard laboratory conditions at the Vivarium of the Institute of Biomedical Research, Medical Faculty, Niš, Serbia. Laboratory was kept under standard temperature (22°C) and humidity (60%), with equal duration of light/dark cycle. During the entire experiment, animals had free access to food and water. All experiments were conducted at the Institute of Biomedical Research, Medical Faculty, Niš, Serbia, and are in accordance with all ethical regulations of European Union (EU Directive of 2010; 2010/63/EU) and Republic of Serbia (332-07-00073/2017-05/1).

#### 2.3.2. Animal Treatment

Treatment protocol included 8 groups of 6 rats treated daily by an intraperitoneal injection (i.p.), as following:

- Control (C) group: the animals were given corn oil (0.2 ml/day) for 10 days.
- Nanoliposomes (NL) group: the animals were given empty nanoliposomes (10 ml/kg) for 10 days.
- Lycopene (LYC) group: the animals were given lycopene dissolved in corn oil (6 mg/kg) for 10 days.
- Encapsulated nanoliposomes (ENL) group: the animals were given encapsulated lycopene (6 mg/kg) for 10 days.
- Methotrexate (MTX) group: the animals were given MTX (20 mg/kg) at day 1 and corn oil (0.2 ml/day) for 10 days.
- Methotrexate-nanoliposomes (MTX-NL) group: the animals were given MTX (20 mg/kg) at day 1 and empty nanoliposomes (10 ml/kg) for 10 days.
- Methotrexate-lycopene (MTX-LYC) group: the animals were given MTX (20 mg/kg) at day 1 and lycopene dissolved in corn oil (6 mg/kg) for 10 days.
- Methotrexate-encapsulated nanoliposomes (MTX-ENL) group: the animals were given MTX (20 mg/kg) at day 1 and encapsulated lycopene (6 mg/kg) for 10 days.

Twenty-four hours after administration of the last dose, all animals were sacrificed by ketamine overdose. For biochemical analysis, blood was taken from the aorta and the kidneys were removed postmortem for tissue biochemical (frozen and stored at −80°C) and histopathological (fixated in 10% buffered formalin) studies. Kidney tissue homogenate (10% *w*/*v*) was made in ice-cold distilled water and was centrifugated at 14000 rpm for 10 min (at 4°C) in order to obtain clear supernatant for further analysis.

### 2.4. Biochemical Analysis

#### 2.4.1. Serum Biochemical Parameters Estimation

The collected blood was allowed to clot at room temperature and afterwards centrifugated in order to obtain serum. Kidney functional state was estimated using serum creatinine and urea levels that were assayed by Olympus AU680® Chemistry-Immuno Analyzer.

#### 2.4.2. Determination of Protein Concentration

Protein concentration in kidney tissue homogenates was measured according to Lowry's method, using bovine serum as standard, as described previously [17].

#### 2.4.3. Malondialdehyde Levels Determination

The determination of the extent of tissue lipid peroxidation was based on the amount of formed malondialdehyde (MDA) estimated by a spectrophotometric method (532 nm), Multiskan Ascent (Labsystems, Finland) using thiobarbituric acid reaction [16]. The amount of tissue MDA was expressed as nmol/mg of kidney tissue proteins.

#### 2.4.4. Catalase Activity Determination

Tissue catalase (CAT) activity was determined using hydrogen peroxide (H_2_O_2_) as substrate, where after the incubation period the reaction was stopped by the addition of ammonium molybdate [18]. The absorbance of the reaction was measured at 405 nm and the results were expressed as IU/mg of proteins.

#### 2.4.5. Advanced Oxidized Proteins Determination

The concentration of advanced oxidized proteins products (AOPP) in renal tissue homogenates was determined by a spectrophotometric method previously described in detail [17]. The method is based on the reaction of AOPP with potassium iodide (KI) in an acidic medium. The intensity of the reaction was measured immediately at 340 nm, and the concentrations of AOPP were expressed as *μ*mol/mg of proteins.

#### 2.4.6. Myeloperoxidase Activity Determination

Myeloperoxidase (MPO) activity was determined in kidney tissue homogenates using *o*-phenylenediamine activated with H_2_O_2_ as described previously [19]. Shortly after the incubation, the reaction was stopped with H_2_SO_4_ solution, and optical densities (OD) were determined at 540 nm using a microplate reader. The results are expressed as OD/mg of proteins.

#### 2.4.7. Tissue Nitrite Concentration Determination

The concentration of nitrites present in tissue homogenates was measured using a Griess reagent [20]. The mixture consisting of tissue homogenate and Griess reagent was incubated at room temperature for 10 min, and the absorbance of each sample was measured at 540 nm using a microplate reader. The nitrite concentrations were calculated using a standard curve of sodium nitrite.

### 2.5. Histopathological Analysis

After fixation of kidney samples in buffered formaldehyde solution (10%, *w/v*), the tissues were routinely processed and embedded in paraffin. Embedded tissue was cut into 4-5 *μ*m thick sections, stained with hematoxylin and eosin (H&E) and periodic acid-Schiff (PAS) as described previously [21] and analyzed using Olympus BH2 light microscope. The extent of kidney tissue damage was evaluated based on morphological changes in sections stained with H&E. The PAS staining allowed a better insight into glomerular and tubular structures appearance and the detection of PAS-positive tubular casts. Semiquantitative scoring system for tubular, interstitial, and glomerular changes was used in order to evaluate the extent of kidney tissue damage. The evaluation criteria were as follows: none (—), mild (+), moderate (++), and severe (+++) [22].

### 2.6. Statistical Analysis

The results were expressed as the mean values ± standard deviation (SD). One-way analysis of variance (ANOVA) followed by Tukey's post hoc test for multiple comparisons (GraphPad Prism version 5.03, San Diego, CA, USA) was used for the determination of statistically significant differences. Probability values (*p*) ≤ 0.05 were considered to be statistically significant.

## 3. Results

### 3.1. Efficacy of Lycopene Encapsulation

The efficacy of encapsulation was 71.9%.

### 3.2. Evaluation of Encapsulated Lycopene Stability

#### 3.2.1. Sustained Release of Encapsulated Lycopene and Susceptibility of Encapsulated Lycopene to H_2_O_2_

**Figure 1:**
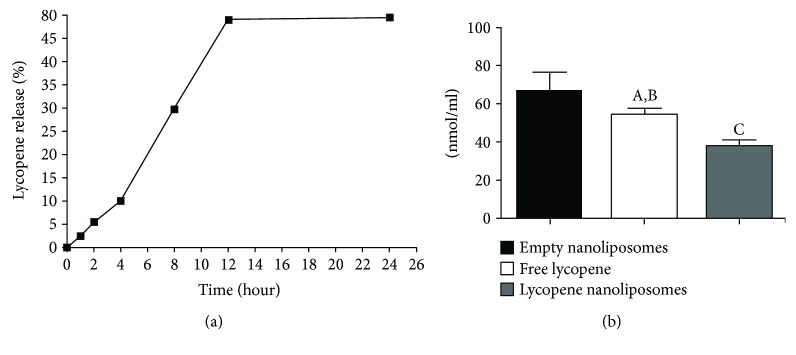
Encapsulated lycopene sustained release curve (a) and MDA levels (b) after exposure of free lycopene, empty nanoliposomes, and encapsulated lycopene nanoliposomes to oxidative damage by incubation with H_2_O_2_. Data are presented as mean value ± SD. ^A^*p* < 0.05 versus empty nanoliposomes; ^B^*p* < 0.01 versus lycopene nanoliposomes; ^C^*p* < 0.001 versus empty nanoliposomes.

The lycopene sustained release curve showed linear growth over 8 h period and stabilization around 12 h, where only 5% of lycopene was released from the nanoliposomes after 2 h and about 50% after 12 h period (Figure 1(a)).

The determined MDA levels after incubation of free lycopene, empty nanoliposomes, and lycopene nanoliposomes with H_2_O_2_ showed that encapsulation of lycopene increases its antioxidant capacity (Figure 1(b)). Lycopene nanoliposomes showed enhanced antioxidant activity, where the concentrations of MDA were significantly decreased in the aliquots of encapsulated nanoliposomes compared to the free lycopene (*p* < 0.01) and empty nanoliposomes (*p* < 0.001) (Figure 1(b)).

#### 3.2.2. pH Dependent Stability of Encapsulated Lycopene

The degradation of lycopene (both free and encapsulated) was the slowest in low pH solutions (6.4 and 7.4) where after 180 min the residual rate of encapsulated lycopene was more than 50% (Table 1). The rapid degradation of free lycopene was observed particularly in solution with pH 9, where residual rate of lycopene after 20 min was 10.4 ± 0.1% and after 180 min 4.1 ± 0.1%. The degradation of encapsulated lycopene in nanoliposomes was slow, and in the first 60 min, the residual rate of lycopene was very high, for example, 60 min after incubation in solution with pH 9 approximately 62% of lycopene remained (Table 1).

#### 3.2.3. Metal Ion Chelating Properties of Encapsulated Lycopene

As shown in Table 2, all metals were chelated more intensively with free lycopene compared to the encapsulated one. The lycopene residual rate in mixtures of K^+^, Ca^2+^, Mg^2+^, Al^3+^, and Cu^2+^ ions and free/encapsulated lycopene was around 35/45, 38/51, 24/38, 24/36, and 33/48%, respectively (Table 2).

### 3.3. In Vivo Evaluation of Free and Encapsulated Lycopene Nephroprotective Activity

#### 3.3.1. Serum Biochemical Parameters Estimation

**Figure 2:**
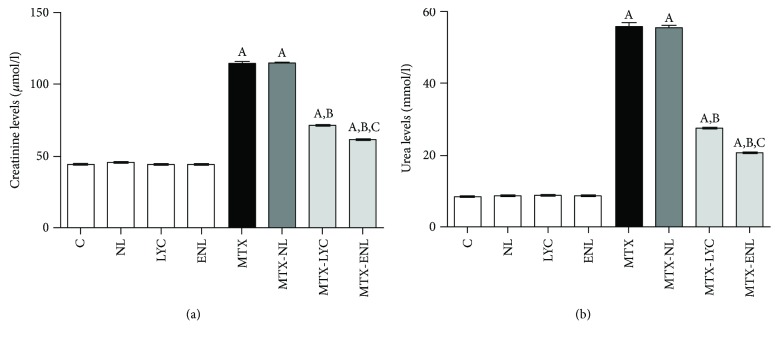
Statistical comparison of serum levels of creatinine (a) and urea (b) between groups of animals after different experimental treatments. Data are presented as mean ± SD, *n* = 6. ^A^*p* < 0.001 versus control group; ^B^*p* < 0.001 versus methotrexate group; ^C^*p* < 0.001 versus methotrexate-lycopene group.

Serum levels of creatinine and urea were statistically significantly increased in animals treated with MTX (Figure 2). The increase in serum levels of these two parameters, especially the levels of urea, following MTX injection, was statistically significantly decreased (*p* < 0.001 compared to MTX-treated group) with the application of lycopene and encapsulated lycopene (Figure 2). Levels of creatinine and urea were statistically significantly decreased in MTX-ENL group compared to MTX-LYC (*p* < 0.001) (Figure 2).

#### 3.3.2. Histopathological Analysis

**Figure 3:**
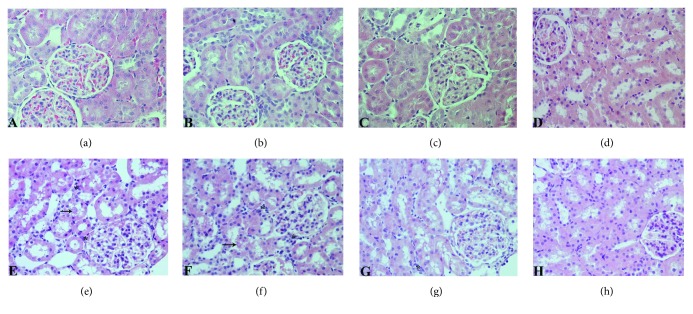
Histological evaluation of renal tissue sections stained with H&E (original magnification, 400x). Sections from control (c) group (a), nanoliposomes (NL) group (b), lycopene (LYC) group (c), and encapsulated nanoliposomes (ENL) group (d) showing normal kidney morphology; (e) methotrexate (MTX) and (f) methotrexate-empty nanoliposomes (MTX-NL) groups with significant damage of renal cortex structures that include glomerular degeneration and interstitial inflammatory infiltration (marked with asterisk) with tubule cell vacuolation and apoptosis and with occasional tubule cast present (marked with arrow); (g) methotrexate-lycopene (MTX-LYC) group showing significantly milder kidney structure changes and (h) methotrexate-encapsulated nanoliposomes (MTX-ENL) group showing higher degree of recovery than those treated with free lycopene form.

Kidney sections of C, NL, LYC, and ENL groups demonstrated normal morphology of rat kidney, with no pathologic findings in glomeruli, tubules, and interstitium (Figures 3(a)–3(d), Table 3). Group of animals treated with MTX showed marked histological damage of renal cortex where the epithelium of proximal tubules showed severe vacuolation and desquamation, with focal tubular necrosis and apoptotic body formation (Figure 3(e), Table 3). In the same group of animals, cast formation was observed in significant proportion of kidney tubules. Glomeruli displayed mild degeneration and edema, with narrowed Bowman's space. There was a significant congestion in glomerular and peritubular capillaries, with mild to moderate interstitial inflammatory infiltrate. In MTX-NL group, micromorphologic changes similar to the MTX group were found (Figure 3(f), Table 3). In the kidneys of animals treated with lycopene after a single dose of MTX, only moderate tubular degeneration, with less prominent vacuolation and desquamation of epithelial cells, was visible (Figure 3(g), Table 3). Tubular cell necrosis was scarce and only occasional tubular casts could be seen. Glomeruli were slightly edematous in appearance, and in interstitium, only mild congestion and with the presence of few inflammatory cells were detected (Figure 3(g), Table 3). In the MTX-ENL group, tubular damage was mildly expressed, with rare small tubular casts (Figure 3(h), Table 3). Glomerular degeneration was not present and the architecture of glomeruli and Bowman's space was well preserved. Interstitial edema and inflammation were unremarkable (Figure 3(h), Table 3).

**Figure 4:**
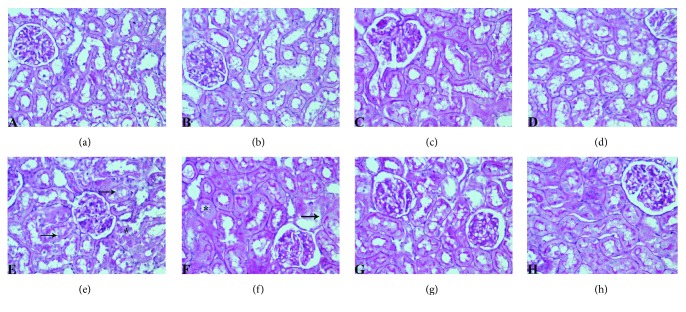
Light photomicrographs of PAS-stained sections of rat renal cortex showing normal histology of kidney tissue and regular morphology of microvilli-covered epithelial surface, with thin and well-defined basement membranes in control (c) group (a) nanoliposomes (NL) group (b), lycopene (LYC) group (c), and encapsulated nanoliposomes (ENL) group (d); significant destruction of brush border (marked with arrow), and presence of PAS-positive tubular casts (marked with asterisk) in (e) methotrexate (MTX) and (f) methotrexate-empty nanoliposomes (MTX-NL) group; attenuation of visible changes, with less destruction of brush in rats treated with methotrexate and lycopene (MTX-LYC group) (g) and almost complete prevention of histopathological alterations in group treated with methotrexate and encapsulated nanoliposomes (MTX-ENL group) (h). (PAS, original magnification, 400x).

PAS-positive epithelial surface, which could be related to microvilli, with thin and well-defined basement membranes of glomerular capillaries and renal tubules, could be seen in the kidney sections of C, NL, LYC, and ENL group of animals (Figures 4(a)–4(d)). In the MTX and MTX-NL groups, loss of brush border in the majority of proximal tubule epithelium, as well as prominent PAS-positive tubular casts, was found (Figures 4(e) and 4(f), Table 3). Several glomeruli matrix expansion was observed as well (Figures 4(e) and 4(f)). Simultaneous treatment with MTX and free lycopene showed significant attenuation of noticeable changes, with less deterioration in brush border and only occasional PAS-positive tubular casts. In the MTX-ENL group, epithelial surface structures were predominantly preserved from MTX toxic damage. Fine tubular brush border and membrane structures were found, and there were no significant changes of glomerular morphology (Figures 4(g) and 4(h), Table 3).

#### 3.3.3. Catalase Activity Determination

Methotrexate application to rats produced a statistically significant decrease in CAT activity in kidney tissue homogenates (*p* < 0.001) (Table 4). The decrease in CAT activity following MTX application was prevented by a prolonged application of both free and encapsulated lycopene (Table 4). The encapsulation of lycopene was found to statistically significantly (*p* < 0.001) enhance the activity of lycopene (Table 4).

#### 3.3.4. Myeloperoxidase Activity Determination

Application of MTX statistically significantly increases MPO activity in kidney tissue compared to the control group of animals (*p* < 0.001) (Table 4). The activity of MPO in the kidneys of animals from MTX-LYC and MTX-ENL groups was statically significantly decreased compared to the MTX-treated animals (*p* < 0.001) (Table 4).

#### 3.3.5. Advanced Oxidized Protein Determination

The extent of protein oxidation in kidney tissues of rats treated with MTX was statistically significantly increased compared to the control group (*p* < 0.001) (Table 4). Ten days of animal treatment, following methotrexate injection, with free or encapsulated lycopene, statistically significantly decreased AOPP formation in the kidneys (*p* < 0.001) (Table 4). The encapsulated lycopene treatment that followed MTX administration completely abolished the formation of AOPP (no statistically significant difference between this group and control group of animals).

#### 3.3.6. Tissue Nitrite Concentration Determination

Application of a single dose of MTX to rats caused a statistically significant increase in renal tissue nitric oxide (NO) concentration, compared to control animals (Table 4). This increase was ameliorated by both free and encapsulated lycopene (Table 4), where the effect of the encapsulated lycopene was statistically significantly higher (*p* < 0.001) than that of the free one (Table 4).

#### 3.3.7. Malondialdehyde Level Determination

The applied dose of MTX produced statistically significant increase in amount of MDA, compared to the control group (*p* < 0.001) (Table 4). These significant changes in MDA products were less pronounced in animals treated with MTX and lycopene and even less in MTX and encapsulated lycopene-treated ones (*p* < 0.001) (Table 4). Also, there is a statistically significant difference (*p* < 0.05) in MDA levels between groups that received lycopene and encapsulated lycopene after MTX (Table 4).

## 4. Discussion

Antioxidants are commonly used as protective agents in numerous pathological conditions; however, their application is often limited due to their low bioavailability, rapid degradation, and delivery of low concentrations to the target tissues. Encapsulation of antioxidants in nanoparticles increases their concentration in cells and antioxidant activity. The initial step, the encapsulation of lycopene, was performed in order to evaluate both its *in vitro* antioxidant activity and *in vivo* protective properties of this highly reactive carotenoid. The efficacy of lycopene encapsulation was determined to be 71.9% which is in accordance with previous investigations (from 60% to 80%) [14]. This range of efficacy of encapsulation can be explained by the strong lipophilicity of lycopene molecules, allowing them to be positioned deeply in bilayer hydrophobic core of nanoliposomes [23]. Our results showed slow release of lycopene from nanoliposomes which reflects its good stability and it can be explained by the fact that lycopene is positioned inside the nanoliposome bilayer. Good sustained release might reflect the potential interaction of free lycopene with plasma proteins and could prolong the presence of lycopene in circulation, allowing it to reach the target tissue.

Since the lycopene has the ability to quench singlet oxygen, which is related to its double bonds in molecule and the opening of the *β*-ionone ring, we examined and compared antioxidant activity of both free and encapsulated lycopene in *in vitro* oxidative damage model. Lycopene showed significant antioxidant activity, which is in accordance with the high reactivity of lycopene with singlet oxygen and free radicals [24]; however, in the present study, the effect of encapsulated form was more pronounced than the activity of free lycopene (Figure 1(b)). Our results are similar to those of Tan et al. [14], where it was showed that lower concentrations of lycopene in nanoliposomes preserved integrity of liposomal membrane and displayed antioxidant activity in lipid peroxidation assays. One can say that the antioxidant activity of lycopene was not only related to its chemical structure, but also to its incorporation in nanoliposomes, where the encapsulated lycopene was able to significantly counteract H_2_O_2_-induced oxidative stress.

It was previously documented that lycopene is rather stable at low pH values, that is, optimal pH range of lycopene is 3.5–4.5 [24], and that its stability decreases with the increase in solutions pH. Having that in mind, we examined whether the encapsulation of lycopene in nanoliposomes can increase its stability in various alkaline solutions with pH varying from 6.4 to 9. The results presented in Table 1 demonstrate the stability of free and encapsulated lycopene in solutions with different pH values where lycopene stability was enhanced by its encapsulation in nanoliposomes. Free lycopene was proven to be less stable than the encapsulated one in alkaline pH, which is in accordance with previous studies [25]. This increased stability of encapsulated lycopene can possibly be explained by its position in the nanoliposomes lipid bilayer, where the polar lycopene is deep in hydrophobic core where it is protected from direct contact with the solution.

Also, we examined the stability of free and lycopene encapsulated in nanoliposomes in the presence of different metal ions (Table 2). The chelation activity of lycopene could reduce its stability and pharmacological activity; thus, by its encapsulation in nanoliposomes, the stability of lycopene against metal ions was improved. This decrease in lycopene chelation after the encapsulation could possibly be related to its higher stability in nanoliposomes and prevention of direct contact of lycopene and metal ions by nanoliposomes membrane.

In *in vivo* experiment, an increase in serum levels of creatinine and urea is direct consequence of kidney tissue injury, a known side effect of MTX application [26]. The changes in kidney tissue following MTX exposure include decrease in glomerular filtration rate, due to vasoconstriction of afferent arterioles, and direct damage of the tubule cells [27]. The increased concentration of MTX in urine, achieved by glomerular filtration and tubular secretion, leads to intratubular crystal formation, causing obstruction [27]. Also an additional mechanism of MTX nephrotoxicity, considered to be key mechanism of toxicity, involves the increase in ROS generation, especially H_2_O_2_, in kidney tubules [28]. Results of biochemical analysis were in accordance with histopathological findings. MTX toxicity may also result in acute renal failure due to precipitation of its metabolite 7-OH-MTX in renal tubules and MTX direct damage of tubules as well [29], which was observed as increased amount of PAS-positive tubular casts (Figure 4(e), Table 3). In the kidneys of animals treated with encapsulated lycopene after MTX, only mild degenerative changes were observed (Table 3), pointing to its strong nephroprotective potential.

Since there is relation between MTX application and oxidative stress, we examined parameters such as CAT, AOPP, MDA, NO, and MPO in kidney homogenates and investigated whether application of free lycopene and nanoliposomes encapsulated with lycopene can improve oxidative status. Catalase is one of the key enzymes that regulate amounts of H_2_O_2_ in cells and indirectly the extent of tissue damage originating from cell structure peroxidation. The decrease (inhibition) in CAT activity could be caused directly by MTX [30], which seems logical since it is proven that methotrexate increases H_2_O_2_ amounts [28]. Also, CAT activity is tightly connected with the amounts of H_2_O_2_ that can cause both inactivation and/or consumption of CAT in kidney tissue [28]. Thus, we can speculate that lycopene, either free or encapsulated, increases kidney CAT activity. This is in good correlation with previous publications where lycopene increased enzymatic antioxidant status (CAT, glutathione S-transferase, peroxidase, and reductase) of liver tissue in animals treated with high-fat diet that causes tissue oxidative damage [31]. Myeloperoxidase activity represents a sensitive marker of tissue infiltration with neutrophils, due to a good correlation between its activity and histopathological finding [32]. Methotrexate is also known to induce the releases of free radicals, MPO among them, from stimulated polymorphonuclear neutrophils causing cellular oxidative damage [28, 32]. Generated products of MPO, chlorinating oxidants arriving from chlorides and H_2_O_2_, are able to cause cell damage by reacting with amino acids, proteins, carbohydrates, lipids, and nucleobases [32]. The application of both forms of lycopene statistically significant decreases MPO activity (Table 4), which is in accordance with previous studies where it is shown that lycopene decreases MPO activity in the damaged kidney [33, 34] and myocardial [35] and pancreatic [36] tissues.

Nitric oxide represents an important cell mediator that is involved in numerous both physiological and pathophysiological processes [37]. One of the theories explaining MTX nephrotoxicity involves NO signaling and increase of inducible nitric oxide synthase (iNOS), which is barely detectable in normal renal tissue [38]. Nitric oxide is also involved in macrophage activation, and during the inflammatory processes in the kidneys, macrophages produce large amounts of NO playing an important role in renal tissue pathology [39]. Previous studies evaluated the effects of free lycopene (10 mg/kg, orally applied) on MTX-induced kidney damage and consequential NO increase [38]. However, no statistically significant effects on NO concentrations were detected. On the other hand, in the model of unilateral ureteral obstruction-induced kidney damage, lycopene was proven to reduce tissue NO levels [33], similarly to the findings of the present study (Table 4). The difference in the obtained results may lay in the different routes of lycopene administration (i.p. and per os), and these results could possibly be explained through high reactivity of lycopene, whose activity significantly decreases when applied orally due to its interaction with numerous compounds leading to its isomerization, oxidation, and/or degradation [14]. In the present study, this was potentially avoided by the application of lycopene via i.p. route and by its encapsulation with nanoliposomes.

The formation of AOPP is believed to be one of the initial steps in cell oxidative stress that further signals the development of renal diseases [40]. Activated MPO in the tissue generates hypochlorous acid which mediates protein modifications causing tyrosine chlorination and formation of chloramines and carbonyls [32]. The accumulation of AOPP in renal cortex, especially in glomerulus, is known to decrease the Bowman's spaces and thus causing the impairments in kidney function [41]. Such pathological findings, as well as increase in AOPP concentration, were detected in animals treated with MTX (Figure 4(e) and Table 4). In addition to ROS-mediated protein damage during exposure to MTX, generated ROS damages unsaturated lipids as well [42], making MDA products a sensitive marker of lipid peroxidation. The formation of MDA could be connected with damaging activity of both peroxynitrite, from superoxide anion and nitric oxide, and hypochlorous acid [28, 32]. Since the activity of MPO and NO concentrations is statistically significantly decreased by both forms of lycopene, it is not surprising that the MDA and AOPP amounts are statistically significantly decreased in these groups as well (Table 4).

This study primarily aimed at comparing efficacy of two different pharmaceutical forms of lycopene on MTX-induced toxicity at kidney tissue level. However, it is limited in defining potential differences of their sites of action at particular kidney tissue cell targets. Previous *in vitro* studies revealed that lycopene can accumulate in epithelial cells and affect different protein synthesis depending on concentration applied [43]. The present study should warrant future work focusing on more detailed action of nanoliposome-encapsulated lycopene at the cellular level.

## 5. Conclusion

Lycopene is presumed to protect cells from oxidative damage by stabilization of the membrane and/or scavenging free radicals generated within the tissue [44]. In this study, we demonstrated that nanoliposome-encapsulated lycopene represents a protected form of this highly reactive carotenoid with enhanced activity. The methotrexate-induced nephrotoxicity model allowed us to investigate and compare the efficacy of the free and encapsulated lycopene in preventing reactive oxygen species-induced kidney tissue damage, which is considered to be a key mechanism of methotrexate toxicity. Our results demonstrated that encapsulated lycopene showed stronger antioxidant activity than free one. Since lycopene is widely used as a dietary supplement, encapsulation of lycopene should be taken as a potential “shelter” when considering lycopene formulation. Also, in this form, it should be recommended for simultaneous application with methotrexate therapy.

## Figures and Tables

**Table 1:** Stability of lycopene nanoliposomes in solutions with different pH values.

pH value	Samples	Residual rate (%) at different time points (minutes)					
		0	20	40	60	120	180
6.4	Free lycopene	62.2 ± 0.6	42.4 ± 1.3	36.4 ± 1.8	33.9 ± 0.9	19.9 ± 1.1	9.0 ± 0.6
	Lycopene nanoliposomes	97.4 ± 0.3	88.4 ± 1.1	75.5 ± 0.3	75.0 ± 0.5	68.4 ± 1.4	58.2 ± 0.4

7.4	Free lycopene	75.15 ± 0.03	32.3 ± 0.6	22.8 ± 0.9	22.1 ± 1.6	16.1 ± 0. 8	4.2 ± 0.3
	Lycopene nanoliposomes	100 ± 0.0	74.2 ± 1.7	60.2 ± 1.9	59.6 ± 0.4	58.6 ± 0.9	53.9 ± 0.0

8	Free lycopene	66.4 ± 0.0	32.1 ± 1.6	25.1 ± 0.9	22.0 ± 0.6	9.5 ± 0.7	3.6 ± 0.3
	Lycopene nanoliposomes	100 ± 0.00	57.6 ± 0.1	53.8 ± 0.6	52.9 ± 0.1	45.5 ± 1.48	44.4 ± 0.2

9	Free lycopene	49.6 ± 0.141	10.4 ± 0.1	10.12 ± 0.2	6.5 ± 0.2	5.65 ± 0.2	4.1 ± 0.1
	Lycopene nanoliposomes	100 ± 0.00	94.9 ± 1.4	87.2 ± 1.1	62.1 ± 0.1	23.2 ± 1.5	20.9 ± 0.177

Data are presented as mean percentage values ± SD.

**Table 2:** Stability of lycopene nanoliposomes against metal ions.

Metal ions	K^+^	Ca^2+^	Mg^2+^	Al^3+^	Cu^2+^
Free lycopene (lycopene residual rate %)	34.75 ± 1.22	38.5 ± 1.64	24.25 ± 0.7	23.75 ± 0.66	33.25 ± 1.05
Lycopene nanoliposomes (lycopene residual rate %)	45.3 ± 2.21	51.22 ± 2.89	38.33 ± 1.69	36.24 ± 1.21	48.1 ± 2.07

Data are presented as mean value ± SD.

**Table 3:** Semiquantitative histopathological score analysis of renal tissue damage.

Histopathological lesion	C	NL	LYC	ENL	MTX	MTX-NL	MTX-LYC	MTX-ENL
*Tubular changes*								
Tubular degeneration	—	—	—	—	+++	+++	++	+
Tubular cell edema/vacuolation	—	—	—	—	+++	+++	++	+
Tubular apoptosis/necrosis	—	—	—	—	++	++	+	—
Hyaline intratubular casts	—	—	—	—	++	++	+	—
*Interstitial changes*								
Mononuclear cell infiltration	—	—	—	—	+++	+++	+	+
Interstitial swelling	—	—	—	—	+++	+++	++	+
Vascular congestion	—	—	—	—	+++	++	++	+
*Glomerular changes*								
Glomerular degeneration	—	—	—	—	++	++	+	—

Scoring was done as follows: none (—), mild (+), moderate (++), and severe (+++).

**Table 4:** Kidney oxidative status in animals after different experimental treatments.

Group/parameter	CAT (IU/mg proteins)	MPO (OD/mg proteins)	AOPP (*μ*mol/mg proteins)	MDA (nmol/mg proteins)	NO (*μ*mol/l)
Control (corn oil treated)	120 ± 2.1	111 ± 4.6	13.5 ± 4.9	10.2 ± 0.6	26.5 ± 5.7
Lycopene	115 ± 3.2	99.5 ± 21.9	12.9 ± 4.3	11.2 ± 1.9	29.5 ± 10.0
Empty nanoliposomes	114 ± 1.3	117.5 ± 6.3	14.2 ± 0.9	11.3 ± 1.4	31.6 ± 7.8
Lycopene nanoliposomes	120 ± 3.5	119 ± 1.4	12.9 ± 1.6	9.9 ± 0.5	30.6 ± 2.8
Methotrexate	24.5 ± 7.5^a^	189 ± 2.3^a^	29.6 ± 6.1^a^	27.9 ± 0.4^a^	65.6 ± 5.8^a^
Lycopene + methotrexate	67.1 ± 2.1^a,b^	159 ± 11.3^a,d^	21.2 ± 0.4^a,d^	17.8 ± 2.2^a,b^	41.9 ± 11.5^b,e^
Empty nanoliposomes + methotrexate	30 ± 4.3^a^	170 ± 13.8^a^	28.9 ± 2.6^e^	25.9 ± 1.6^a,^	66.3 ± 3.5 ^a^
Lycopene nanoliposomes + methotrexate	83.1 ± 3.2^a,b,c^	141.5 ± 14^a,b^	17.9 ± 1.8^b^	14.6 ± 2.4^a,b,f^	39.3 ± 1.4^b,c,e^

^a^ *p* < 0.001 versus control group treated with corn oil; ^b^*p* < 0.001 versus methotrexate group; ^c^*p* < 0.001 versus lycopene + methotrexate group; ^d^*p* < 0.01 versus methotrexate group; ^e^*p* < 0.05 versus control group treated with corn oil; ^f^*p* < 0.05 versus lycopene + methotrexate group

## References

[1] Widemann B. C., Balis F. M., Kempf-Bielack B. (2004). High-dose methotrexate-induced nephrotoxicity in patients with osteosarcoma. *Cancer*.

[2] Kivity S., Zafrir Y., Loebstein R., Pauzner R., Mouallem M., Mayan H. (2014). Clinical characteristics and risk factors for low dose methotrexate toxicity: a cohort of 28 patients. *Autoimmunity Reviews*.

[3] Jahovic N., Sener G., Cevik H., Ersoy Y., Arbak S., Yegen B. C. (2004). Amelioration of methotrexate-induced enteritis by melatonin in rats. *Cell Biochemistry & Function*.

[4] Asvadi I., Hajipour B., Asvadi A., Asl N. A., Roshangar L., Khodadadi A. (2011). Protective effect of pentoxyfilline in renal toxicity after methotrexate administration. *European Review for Medical and Pharmacological Sciences*.

[5] Vardi N., Parlakpinar H., Ates B., Cetin A., Otlu A. (2013). The protective effects of *Prunus armeniaca* L (apricot) against methotrexate-induced oxidative damage and apoptosis in rat kidney. *Journal of Physiology and Biochemistry*.

[6] Palabiyik S. S., Erkekoglu P., Zeybek N. D. (2013). Protective effect of lycopene against ochratoxin A induced renal oxidative stress and apoptosis in rats. *Experimental and Toxicologic Pathology*.

[7] Stahl W., Sies H. (2003). Antioxidant activity of carotenoids. *Molecular Aspects of Medicine*.

[8] Atessahin A., Yilmaz S., Karahan I., Ceribasi A. O., Karaoglu A. (2005). Effects of lycopene against cisplatin-induced nephrotoxicity and oxidative stress in rats. *Toxicology*.

[9] Dogukan A., Tuzcu M., Agca C. A. (2011). A tomato lycopene complex protects the kidney from cisplatin-induced injury via affecting oxidative stress as well as Bax, Bcl-2, and HSPs expression. *Nutrition and Cancer*.

[10] Sahoo S. K., Parveen S., Panda J. J. (2007). The present and future of nanotechnology in human health care. *Nanomedicine: Nanotechnology, Biology, and Medicine*.

[11] Hughes G. A. (2005). Nanostructure-mediated drug delivery. *Nanomedicine: Nanotechnology, Biology, and Medicine*.

[12] Mozafari M. R., Mozafari M. R. (2005). Bioactive entrapment and targeting using nanocarrier technologies: an introduction. *Nanocarrier Technologies: Frontiers of Nanotherapy*.

[13] Kocic G., Tomovic K., Kocic H. (2017). Antioxidative, membrane protective and antiapoptotic effects of melatonin, *in silico* study of physico-chemical profile and efficiency of nanoliposome delivery compared to betaine. *RSC Advances*.

[14] Tan C., Xue J., Abbas S., Feng B., Zhang X., Xia S. (2014). Liposome as a delivery system for carotenoids: comparative antioxidant activity of carotenoids as measured by ferric reducing antioxidant power, DPPH assay and lipid peroxidation. *Journal of Agricultural and Food Chemistry*.

[15] Chen X., Zou L. Q., Niu J., Liu W., Peng S. F., Liu C. M. (2015). The stability, sustained release and cellular antioxidant activity of curcumin nanoliposomes. *Molecules*.

[16] Buege J. A., Aust S. D. (1978). Microsomal lipid peroxidation. *Methods in Enzymology*.

[17] Ilic S., Stojiljkovic N., Veljkovic S. (2016). Morphometric study of structural kidney damages caused by cisplatin in rats. Effects of quercetin. *Acta Microscopica*.

[18] Goth L. (1991). A simple method for determination of serum catalase activity and revision of reference range. *Clinica Chimica Acta*.

[19] Jacob S. S., Shastry P., Sudhakaran P. R. (2001). Influence of non-enzymatically glycated collagen on monocyte–macrophage differentiation. *Atherosclerosis*.

[20] Radulovic N. S., Randjelovic P. J., Stojanovic N. M., Cakic N. D., Bogdanovic G. A., Zivanovic A. V. (2015). Aboriginal bush foods: a major phloroglucinol from Crimson Bottlebrush flowers (*Callistemon citrinus*, Myrtaceae) displays strong antinociceptive and antiinflammatory activity. *Food Research International*.

[21] Stojiljkovic N., Stoiljkovic M., Randjelovic P., Veljkovic S., Mihailovic D. (2012). Cytoprotective effect of vitamin C against gentamicin-induced acute kidney injury in rats. *Experimental and Toxicologic Pathology*.

[22] Randjelovic P., Veljkovic S., Stojiljkovic N. (2012). Salicylic acid attenuates gentamicin-induced nephrotoxicity in rats. *The Scientific World Journal*.

[23] van de Ven M., Kattenberg M., van Ginkel G., Levine Y. K. (1984). Study of the orientational ordering of carotenoids in lipid bilayers by resonance-Raman spectroscopy. *Biophysical Journal*.

[24] Anguelova T., Warthesen J. (2000). Degradation of lycopene, *α*-carotene, and *β*-carotene during lipid peroxidation. *Journal of Food Science*.

[25] Salama M. F., Seliem E. I., Mahmoud K. F., Amin A. A. (2015). Physiochemical characterization and oxidative stability of encapsulated nano lycopene pigments extracted by CO_2_ fluid extraction. *International Journal of Current Microbiology and Applied Sciences*.

[26] Singh R., Shah R., Turner C., Regueira O., Vasylyeva T. L. (2015). N-acetylcysteine renoprotection in methotrexate induced nephrotoxicity and its effects on B-cell lymphoma. *Indian Journal of Medical and Paediatric Oncology*.

[27] Perazella M. A. (1999). Crystal-induced acute renal failure. *The American Journal of Medicine*.

[28] Ahmed W., Zaki A., Nabil T. (2015). Prevention of methotrexate-induced nephrotoxicity by concomitant administration of garlic aqueous extract in rat. *Turkish Journal of Medical Sciences*.

[29] Widemann B. C., Adamson P. C. (2006). Understanding and managing methotrexate nephrotoxicity. *The Oncologist*.

[30] Coleshowers C. L., Oguntibeju O. O., Ukpong M., Truter E. J. (2010). Effects of methotrexate on antioxidant enzyme status in a rodent model. *Medical Technology SA*.

[31] Choi S. K., Seo J. S. (2013). Lycopene supplementation suppresses oxidative stress induced by a high fat diet in gerbils. *Nutrition Research and Practice*.

[32] Kolli V. K., Abraham P., Isaac B., Selvakumar D. (2009). Neutrophil infiltration and oxidative stress may play a critical role in methotrexate-induced renal damage. *Chemotherapy*.

[33] Atilgan H. I., Aydin A., Sadic M. (2016). The protective effect of lycopene on kidney against experimentally induced unilateral ureteral obstruction. *Acta Medica Mediterranea*.

[34] Sadic M., Atilgan H. I., Aydin A. (2017). Scintigraphic, histopathologic, and biochemical evaluation of lycopene effects on renal ischemia reperfusion injury in rats. *Serbian Archives of Medicine*.

[35] Aman U., Vaibhav P., Balaraman R. (2012). Tomato lycopene attenuates myocardial infarction induced by isoproterenol: electrocardiographic, biochemical and anti-apoptotic study. *Asian Pacific Journal of Tropical Biomedicine*.

[36] Ozkan E., Akyüz C., Dulundu E. (2012). Protective effects of lycopene on cerulein-induced experimental acute pancreatitis in rats. *The Journal of Surgical Research*.

[37] Hou Y. C., Janczuk A., Wang P. G. (1999). Current trends in the development of nitric oxide donors. *Current Pharmaceutical Design*.

[38] Oguz E., Kocarslan S., Tabur S., Sezen H., Yilmaz Z., Aksoy N. (2015). Effects of lycopene alone or combined with melatonin on methotrexate-induced nephrotoxicity in rats. *Asian Pacific Journal of Cancer Prevention*.

[39] Rolle U., Shima H., Puri P. (2002). Nitric oxide, enhanced by macrophage-colony stimulating factor, mediates renal damage in reflux nephropathy. *Kidney International*.

[40] Yamagishi S., Matsui T. (2010). Advanced glycation end products, oxidative stress and diabetic nephropathy. *Oxidative Medicine and Cellular Longevity*.

[41] Liu Y. (2009). Advanced oxidation protein products: a causative link between oxidative stress and podocyte depletion. *Kidney International*.

[42] Uz E., Oktem F., Yilmaz H. R., Uzar E., Ozguner F. (2005). The activities of purine-catabolizing enzymes and the level of nitric oxide in rat kidneys subjected to methotrexate: protective effect of caffeic acid phenethyl ester. *Molecular and Cellular Biochemistry*.

[43] Hantz H. L., Young L. F., Martin K. R. (2016). Physiologically attainable concentrations of lycopene induce mitochondrial apoptosis in LNCaP human prostate cancer cells. *Experimental Biology and Medicine*.

[44] Krishnamoorthy G., Selvakumar K., Venkataraman P., Elumalai P., Arunakaran J. (2013). Lycopene supplementation prevents reactive oxygen species mediated apoptosis in Sertoli cells of adult albino rats exposed to polychlorinated biphenyls. *Interdisciplinary Toxicology*.

